# Integrating phylogenies with chronology to assemble the tree of life

**DOI:** 10.3389/fbinf.2025.1571568

**Published:** 2025-04-30

**Authors:** Jose Barba-Montoya, Jack M. Craig, Sudhir Kumar

**Affiliations:** ^1^ Richard Gilder Graduate School, American Museum of Natural History, New York, NY, United States; ^2^ Institute for Genomics and Evolutionary Medicine, Temple University, Philadelphia, PA, United States; ^3^ Department of Biology, Temple University, Philadelphia, PA, United States

**Keywords:** timetree, supertree, tree of life, supermatrix, algorithm

## Abstract

Reconstructing the global Tree of Life necessitates computational approaches to integrate numerous molecular phylogenies with limited species overlap into a comprehensive supertree. Our survey of published literature shows that individual phylogenies are frequently restricted to specific taxonomic groups due to investigators’ expertise and molecular evolutionary considerations, resulting in any given species present in a minuscule fraction of phylogenies. We present a novel approach, called the chronological supertree algorithm (Chrono-STA), that can build a supertree of species from such data by using node ages in published molecular phylogenies scaled to time. Chrono-STA builds a supertree by integrating chronological data from molecular timetrees. It fundamentally differs from existing approaches that generate consensus phylogenies from gene trees with missing taxa, as Chrono-STA does not impute nodal distances, use a guide tree as a backbone, or reduce phylogenies to quartets. Analyses of simulated and empirical datasets show that Chrono-STA can combine taxonomically restricted timetrees with extremely limited species overlap. For such data, approaches that impute missing distances or assemble phylogenetic quartets did not perform well. We conclude that integrating phylogenies via temporal dimension enhances the accuracy of reconstructed supertrees that are also scaled to time.

## 1 Introduction

Reconstructing the history of life on Earth is foundational to studying evolution and biodiversity, which is pursued by many taxonomists, systematists, and evolutionary biologists. Molecular phylogenetics has been a key tool to infer the evolutionary relationships of organisms (Hedges and Kumar, 2009; Yang and Rannala, 2012). Occasionally, large phylogenies are constructed by extensive sampling of species from major groups like birds, squamates, mammals, and fishes (Jetz et al., 2012; Tonini et al., 2016; Hughes et al., 2018; Upham et al., 2019; Álvarez-Carretero et al., 2022). Yet, much more commonly, published phylogenies are the work of taxon specialists who focus on individual families or genera due to their organismal expertise. Furthermore, even considering the increased accessibility of genetic data and improvements in computational power, technical impediments still stand in the way of building large-scale phylogenies. For example, while certain genetic loci contain valuable phylogenetic signals in some taxa, they may be largely invariant or actively misleading in others (Gonçalves et al., 2019). Moreover, teasing apart orthologous from paralogous sequences can be challenging, especially among increasingly distantly related taxa (Koonin, 2005; Altenhoff et al., 2019). Similarly, the best models to capture the processes of sequence evolution in one clade may be inappropriate for another (Fitch, 1971; Lopez et al., 2002; Kumar et al., 2005). Therefore, many small and large taxonomically restricted phylogenies have been published (Hedges et al., 2015; Kumar et al., 2022).

**FIGURE 1:**
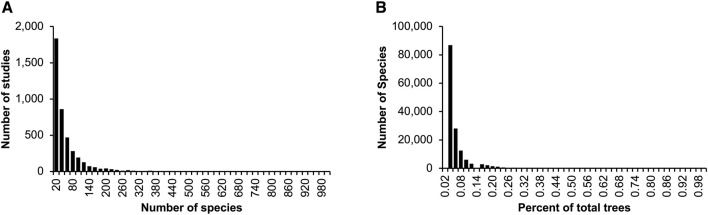
Summary characteristics of published timetrees curated for the TimeTree database (Kumar et al., 2022). Distributions are shown for the **(A)** number of species in phylogenies, **(B)** count of phylogenies in which a species occurs, as a percentage of the total number of trees. These statistics were derived from 4,185 phylogenies published in the last five decades. Only species counts up to 1,000 **(A)** and percentages up to 1% **(B)** are shown.

Fundamental properties of published phylogenies can be gleaned from the collection of more than 4,000 phylogenies curated for the TimeTree database (Kumar et al., 2022). Across the whole collection, phylogenies contained a median of 25 species each (Figure 1A), each found in a median of just one timetree (0.02% of the sample) (Figure 1B). Consequently, the average number of species common between any two phylogenies is less than 1.0.

While many supertree methods exist to unite a collection of phylogenies (e.g., Swenson et al., 2012; Creevey and McInerney, 2005; Morel et al., 2022; Vachaspati and Warnow, 2015; Zhang et al., 2018), they are not often intended to accommodate such limited taxonomic overlap. Some of these tools (e.g., Morel et al., 2022; Vachaspati and Warnow, 2015; Zhang et al., 2018) are typically rooted in coalescent theory and used to reconcile samples of hundreds or thousands of phylogenies derived from a single gene each, as an alternative to concatenating these multi-gene datasets into unified alignment and building a single tree. This is useful in cases where the pattern of inheritance at speciation may differ among genes, leading to conflicting phylogenetic signal (due to processes such as horizontal gene transfer, hybridization, and incomplete lineage sorting), or where the concatenated alignment may have many gaps where a given species lacks molecular data.

**FIGURE 2:**
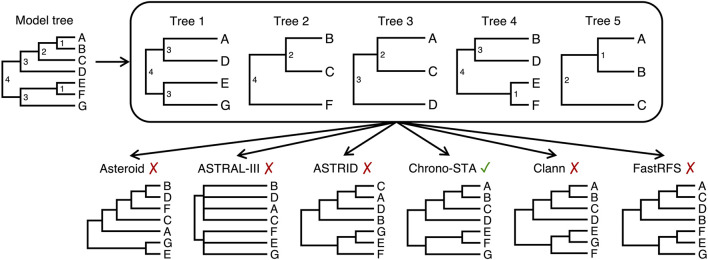
Five timetrees with partial species overlaps, derived from a model tree by randomly sampling taxa (node ages are shown to the right of each node). Supertrees were produced by gene tree reconciliation methods that can handle missing data: Asteroid, Astral-III, ASTRID, Clann, and FastRFS. The new Chrono-STA approach was able to produce the correct supertree using divergence times.

The latter case is particularly true when combining disparate phylogenies, as illustrated in Figure 2, which depicts a collection of five timetrees (trees 1–5) derived from a model tree of seven distinct species (Figure 2, model tree; species A to G). Five existing supertree methods were applied, each using different strategies to address missing data and reconcile input trees. Methods like Asteroid (Morel et al., 2022) and ASTRID (Vachaspati and Warnow, 2015) impute missing nodal distances between species, while ASTRAL-III (Zhang et al., 2018), decomposes input trees into all possible four-species relationships (quartets) and reconciles these into a consensus topology. In Clann (Creevey and McInerney, 2005), the MSSA (Matrix-based Supertree Scoring Algorithm) scoring method addresses missing data and reconciles input trees by comparing the path length distance matrix derived from a source tree with another distance matrix derived from a pruned supertree. The differences between the matrices are scored, and the sum of the scores from all comparisons is calculated. FastRFS (Vachaspati and Warnow, 2017) constructs supertrees by minimizing the total Robinson-Foulds (RF) distance (Robinson and Foulds, 1981) between the input trees and the resulting supertree. This method handles missing data by computing a set of allowed bipartitions (*X*) from the input trees (which are splits of the leaf set into two parts) each defined by deleting missing edges in the species tree being constructed. The output tree draws its bipartitions from X.

Due to the minimal taxonomic overlap between the input trees, none of them could recover the true topology in this case (Figure 2). This highlights the need for a supertree approach developed specifically for species trees, not gene trees, which can accommodate even the extreme lack of taxonomic overlap observed in the TimeTree database. To solve this problem, we developed a chronological supertree algorithm, Chrono-STA, which was able to unite the provided sample of five trees on the basis of their shared timescale (Figure 2). Chrono-STA uses the provided divergence times among taxa to merge species into a supertree by first connecting the most closely related species (those sharing the shortest divergence time) across all input trees and then repeating this step iteratively. The important advance which makes Chrono-STA more powerful than other clustering approaches is that once each cluster is formed, it is back-propagated to all input trees, improving their information content and increasing the power of each successive cluster inference. Thus, incorporating chronological information fully throughout the tree building process can mitigate the extremely limited and uneven taxonomic overlap observed in empirical timetrees.

Other approaches developed to incorporate chronological information in supertree construction are more limited in scope and capability. First, the TimeTree of Life website has the ability to take a user-provided species list and extract the corresponding subset from their synthetic phylogeny based on more than 150,000 published timetrees, even performing background taxonomic substitutions where needed to increase coverage based on phylogenetic principles (for example, if a user were to request a tree of orangutans, gorillas and humans, but the TimeTree databased lacked humans, it would substitute the divergence time between gorillas and chimpanzees, since phylogenetic principles dictate that any two sister species like humans and chimpanzees are equally closely related to a third species, like gorillas). DateLife (Sánchez Reyes et al., 2024) is another web-based tool and R package which performs this same function, taking either an untimed cladogram or a list of species and returning a timetree derived from the Open Tree of Life (Hinchliff et al., 2015). Importantly, neither of these tools estimates novel divergence times nor do they have the ability to take timetrees as an input and integrate them to broaden an existing phylogenetic consensus.

One tool with this capacity is presented in Hedges et al. (2015). Their hierarchical average linkage (HAL) clustering approach builds supertimetrees using divergence times to resolve polytomies in the NCBI backbone taxonomy, then performs localized branch swapping to make evolutionary relationships maximally consistent with the topologies. While HAL was used to assemble a supertree of more than 148,000 species from published phylogenies (Hedges et al., 2015; Kumar et al., 2022), it is still limited by its requirement of a phylogenetic backbone, which creates many additional polytomies in cases where the sample of input trees conflict with the backbone and one another. For large empirical datasets like the TimeTree database, this is a common problem. Thus, while tools exist to build supertimetrees either *de novo* or by extraction from a comprehensive tree, there is still a need for an algorithm which more elegantly combines timed phylogenies on the basis of their shared phylogenetic scale without the need for a backbone and without inducing additional topological conflict.

We present Chrono-STA, which does not require a phylogenetic backbone to build a supertree from a collection of timetrees, and thus avoids the taxonomic confusion induced by HAL. It pairs species using all the input timetrees analyzed in parallel independently without the need to impute missing nodal distances between taxa, as in some methods (Vachaspati and Warnow, 2015; Morel et al., 2022). In this study, we first introduce the concept and implementation of Chrono-STA, then demonstrate its usage by analyzing both computer-simulated and empirical datasets. In these examples, timetrees have very few common species to mimic the patterns observed in the corpus of published timetrees (Figure 1). We also applied five supertree approaches to these datasets to evaluate the relative performance of different methods for combining phylogenies with partial overlaps.

## 2 Material and methods

### 2.1 A novel chronological supertree approach (Chrono-STA)

**FIGURE 3:**
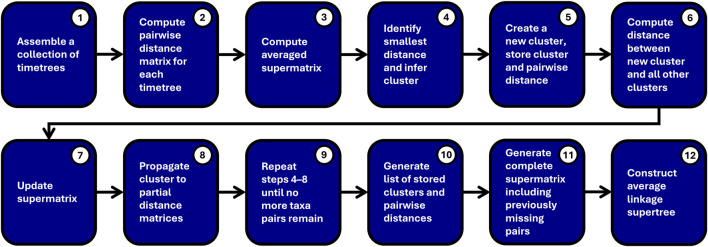
Overview of the steps in the chronological supertree algorithm (Chrono-STA).

Chrono-STA employs a novel approach that builds on classical unweighted pair group method with arithmetic mean (UPGMA; Sokal and Michener, 1958) but incorporates the temporal dimension to build a consensus from a collection of input timetrees even when topological overlap between them is extremely sparse, and even when there exists chronological or topological disagreement between them. Chrono-STA is a supermatrix apprach that utilizes a novel backpropagation of chronologically defined taxa pairs (Figure 3). In a Chrono-STA run, a collection of input timetrees are first decomposed into pairwise distance matrices, and a supermatrix is computed encompassing every taxon found in all input trees. The supermatrix is then populated with pairwise distances between taxa, as defined by the divergence times found in each input timetree. If a given divergence is identified in multiple timetrees, the associated cell in the supermatrix is populated with the mean value across all matching divergences found among input trees. Thus, the initial supermatrix represents the consensus of all divergence time estimates drawn from the collection of input timetrees. Here, a simple average linkage clustering could be carried out, but this is typically not possible due to missing pairwise divergences.

This is where Chrono-STA diverges from conventional supermatrix approaches. We first define the closest sister pair in the initial supermatrix by identifying the lowest divergence time present, and cluster this pair as a new single taxon. But instead of repeating this process in the initial supermatrix, we then backpropagate this newly defined taxon into all the input timetrees, replacing one or both of the two constituent taxa. This has the effect of increasing taxon completeness in all those trees where one, but not both, of the two taxa were found. Additionally, this allows us to define divergence times between the newly added clustered taxon and all species which shared a divergence time with either one or both members of the pair. So, by back-propagating clustered taxa in this way, we use a phylogenetic principle, that any two sister taxa are equally closely related to a third, unrelated.

Having accomplished this backpropagation and information augmentation, we then repeat the process of building a consensus supermatrix from all updated input matrices. The resulting new supermatrix will have one fewer taxon than the initial version, but will have increased in completeness, and we will have defined the first taxon pair and their divergence time. We then repeat this process until the consensus supermatrix is reduced to a final pairwise distance, noting the pair formed and their divergence time at each step, as with a conventional average linkage method.

At this point, we have an ordered list of clustered taxa and their divergence times, which are used to define a timetree, again, using the average linkage approach. We apply time-smoothing to the constructed timetree using non-negative least squares, ensuring that all terminal branches end at time zero. While Chrono-STA is conceptually the same as the classical UPGMA approach, it represents a substantial advance in that we can proceed with our analysis despite large gaps in the data and multiple pairwise time matrices are handled at the same time. Furthermore, we define our divergence times based on much more input data per node, as we gain information by backpropagating clusters and using them to establish new divergence times in the input matrices. Thus, sound phylogenetic principles allow us to overcome the limitations of sparse data and improve the power of our inferences. This method can also be applied to combine trees from partially overlapped multi-sequence alignments (MSAs) by inferring a maximum likelihood (ML) tree for each MSA and dating each ML tree. Then, the timetrees are combined following the outlined procedure.

For clarity, the Chrono-STA method may be represented by the following pseudocode (Figure 3):

1. A collection of timetrees is assembled.
2. A Pairwise time matrix is computed independently for each timetree.
3. These timetree-specific distance matrices are compiled into a cumulative averaged supermatrix.
4. The sister pair *ij* with the smallest distance, $D_{ij}$ , is identified and designated as the first cluster.
5. This cluster, *ij*, which has $n_{\left(ij\right)}=n_{i}+n_{j}$ members, is recorded along with its pairwise distance.
6. The distance between the new cluster and all the other clusters (except for *i* and *j*) is computed by using: $D_{\left(ij\right),k}=\left[\frac{n_{i}}{n_{i}+n_{j}}\right]D_{ik}+\left[\frac{n_{j}}{n_{i}+{\;n}_{j}}\right]D_{jk}$ .
7. The supermatrix is updated by removing the columns and rows corresponding to groups *i* and *j*, then adding a column and row for the cluster (*ij*).
8. Cluster *ij* is propagated back to all partial distance matrices by removing the corresponding columns and rows for groups *i* and *j*, then add a column and row for cluster (*ij*). Their pairwise distance is stored.
9. Steps 4–8 are repeated until no more taxa pairs with divergence times remain.
10. A list of stored clusters and pairwise distances is generated.
11. A complete distance supermatrix including previously missing pairs is generated from this list.
12. A supertree is constructed from this matrix using average linkage.

### 2.2 Tested methods

Using simulated and empirical data, we compared the performance of Chrono-STA and four other supertree construction methods. Chrono-STA requires no fine-tuning parameters for analysis except for the collection of supertrees. For Asteroid (Morel et al., 2022), a heuristic search was conducted to find the supertree with the lowest global induced length. Asteroid begins with a specified supertree topology and utilizes a tree search strategy, incorporating SPR moves to optimize the score. We used 20 randomly generated starting trees (-r 20). The supertree topology was iteratively optimized by an adapted FastME (Lefort et al., 2015) tree search algorithm to the global induced length score. For ASTRAL-III (Zhang et al., 2018), a heuristic search was conducted. Branches on the supertree were scored using the posterior probability for the main resolution (-t 3). The lambda parameter for the Yule prior (Yule, 1925), used branch lengths and posterior probabilities (-c) calculations, was set to 0.5. For ASTRID (Vachaspati and Warnow, 2015), the FastME analysis was conducted with both nearest neighbor interchange (NNI) and subtree-pruning-and-grafting SPR moves (-s option), and (-u) to use UPGMA completion. For Clann (Creevey and McInerney, 2005) we conducted a heuristic search for the best supertree, applying the most similar supertree criterion and subtree pruning and regrafting (SPR) move, with a maximum number of steps (nsteps) set to three, maximum number of swaps (maxswaps) set to 1,000,000, and 10 repetitions of heuristic search, utilizing a weighting scheme of comparisons. Starting trees were derived from neighbor-joining tree from average consensus distances, with missing data estimated using four-point condition distances. FastRFS (Vachaspati and Warnow, 2017) is based on a dynamic programming method to find an exact solution to the RF supertree problem within a constrained search space. ASTRAL was used to compute the allowed bipartitions (*X*), calculate quartet distances, and combine them into clusters using both distance matrix-based and greedy consensus methods. The single optimal tree generated was reported.

#### 2.2.1 Quantifying and comparing performance

The performance of the methods for constructing supertrees was assessed by calculating RF distances between the inferred and reference tree. This calculation is performed using the R function MultiRF (Revell, 2012). The normalized Robinson-Foulds (nRF) distance estimates the topological error in phylogeny reconstruction. It is calculated as nRF = RF/(2(m − 3)), where *m* is the number of species. The model timetree was the reference tree for simulated datasets, whereas the timetree published in the original study was assumed to be the reference tree in the analysis of empirical data. Additionally, we compared the RF distance between published input trees and their relative subset of our inferred Chrono-STA tree by using a polytomy-aware implementation of the RF metric.

Chrono-STA also produces node ages in the inferred supertree, compared with the times in the reference tree. Because the topologies of the inferred and reference phylogenies were not identical, we compared the node times in the reference tree with the most recent common ancestor (MRCA) node times in the Chrono-STA timetree for the sets of taxa included within each node in the reference tree. The slope and coefficient of determination (*R* ^2^) for the linear regression through the origin were computed for the comparison of the inferred supertree and the reference tree. Furthermore, the difference between the estimated MRCA node times and reference tree node times was computed. The difference was divided by the reference tree node time and multiplied by 100 to generate a percent time error (ΔTE).

### 2.3 Datasets

#### 2.3.1 Simulated datasets

**FIGURE 4:**
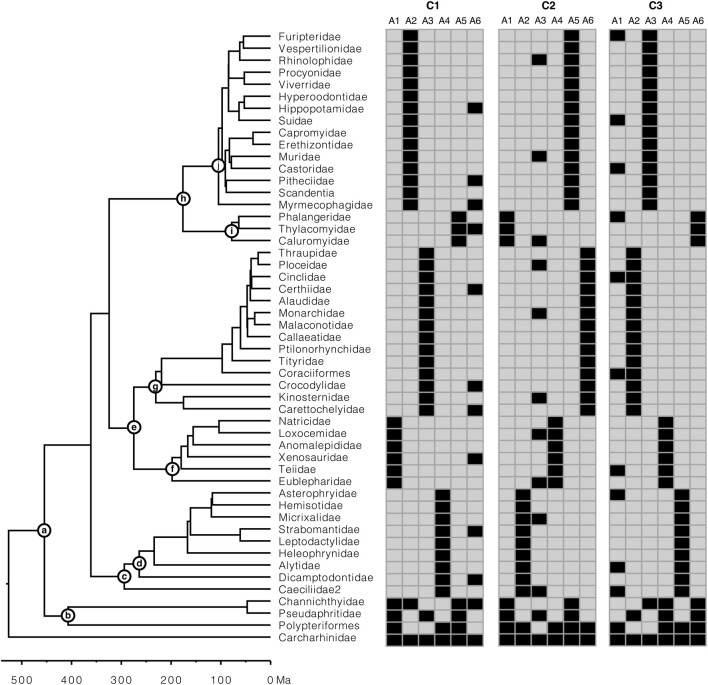
Presence-absence representation of the three simulated collections (C1-C3) of six gene alignments (A1-A6) each, with extremely low overlap of species, generated to construct supertrees from constituent trees. Each collection consists of five constituent timetrees along a backbone timetree. The calibrations (nodes a-j) used to construct the constituent timetrees are represented on the model timetree (reference tree). The rooting outgroup (Carcharhinidae) was excluded from the analysis.

To assess the performance of Chrono-STA in constructing supertrees from timetrees with extremely low species overlap, three small collections (C1-C3) of six timetrees (T1-T6) were generated (Figure 4). Each timetree was derived from an alignment of 51 species from the collection of sequence alignments utilized previously by Tamura et al. (2012). They generated alignments using SeqGen (Rambaut and Grassly, 1997) under the HKY substitution model (Hasegawa et al., 1985) and heterogeneous sets of evolutionary parameters, including sequence lengths (258–9,353 sites), evolutionary rates (ranging from 1.35 to 2.60 substitutions per site per billion years), G + C-content bias (G + C contents ranging from 39% to 82%), and transition/transversion rate bias (transition/transversion ratio, ranging from 1.9 to 6.0. We selected six nucleotide gene alignments (A1-A6; ranging from 2,174 to 3,100 sites) simulated with autocorrelated rate variation among lineage, such that the rate of a descendant branch was drawn from a lognormal distribution centered around the mean rate of the ancestral branch; an autocorrelation parameter *ν* = 1 was used (Kishino et al., 2001). Their original datasets contained 446 species, but we sampled 51 species, as in Barba-Montoya et al. (2023), for practicality (Figure 4). During species down-sampling, an outgroup as well as at least one of the ingroup root taxa selected to ensure that timetrees could be produced from sequence alignments.

Constituent timetrees were inferred using gene alignments for every collection (CI-C3). First, a ML tree was inferred from each gene alignment using the HKY+Γ5 model in IQ-TREE (Nguyen et al., 2015). Then, each ML tree was dated using RelTime (Tamura et al., 2012) in MEGA-CC (Kumar et al., 2012; Tamura et al., 2021). Each timetree was computed using a set of 10 calibrations, each assigning a uniform distribution: (a) U(453, 457 million years ago (Ma)), (b) U(405, 409 Ma), (c) U(292, 296 Ma), (d) U(262, 266 Ma), (e) U(273, 277 Ma), (f) U(196, 200 Ma), (g) U(229, 233 Ma), (h) U(174, 178 Ma), (i) U(76, 80 Ma), and (j) U(103, 107 Ma). The rooting outgroup (Carcharhinidae) was excluded from the analysis because RelTime analysis does not produce estimates in the outgroup (Tamura et al., 2018; Tamura et al., 2012). Each collection contained five taxon-restricted timetrees with limited overlap and one timetree with one or more species per major group. These timetrees were derived from 51-species alignments by a realistic process that ensured that every collection’s individual timetree (T1-T6) contained phylogenetic errors and produced node ages with variance, as would be the case in real studies. The timetrees produced were missing an average of 78% of species, with a range of 67%–88%, and had a limited species overlap.

To evaluate the impact of uncertainty in node times derived from constituent timetrees on the inferred supertree topology, we used timetree collections C1–C3. From each collection, three new collections were generated by increasing the original node time variance of each constituent timetree by one (var 1×), two (var 2×), and three (var 3×) times.

#### 2.3.2 Mammal dataset

**FIGURE 5:**
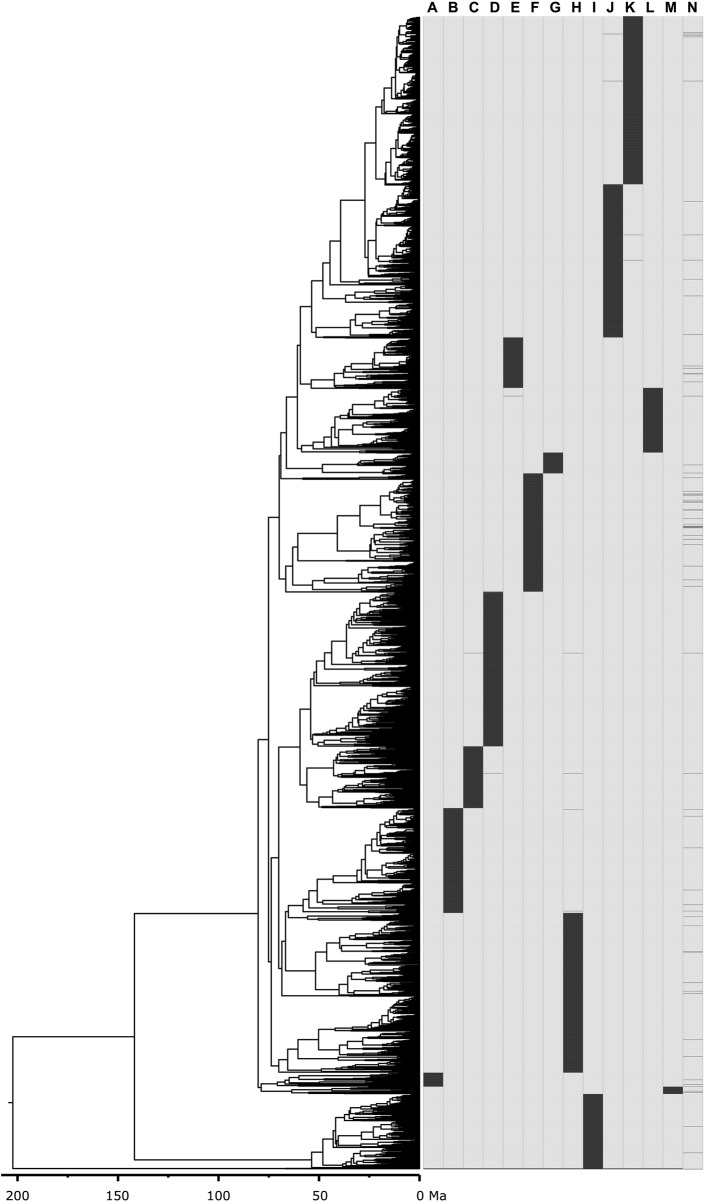
Mammal timetree consisting of 4,705 species across 14 constituent timetrees including a backbone timetree (Álvarez-Carretero et al., 2022). Presence-absence representation of 14 constituent timetrees: **(A)** Afrotheria, **(B)** Artiodactyla, **(C)** Chiroptera_subt1, **(D)** Chiroptera_subt2, **(E)** Ctenohystrica, **(F)** Euarchonta, **(G)** Lagomorpha, **(H)** Laurasiatheria_therest, **(I)** Marsupialia, **(J)** Rodentia_therest_subt1, **(K)** Rodentia_therest_subt2, **(L)** Sciuridae_and_related, **(M)** Xenarthra, **(N)** 00_main_tree_T2-updated-geochronolog (backbone timetree).

To assess the performance of the methods in constructing supertrees from limited overlapping timetrees using empirical data, we used the mammal timetree of Álvarez-Carretero et al. (2022), which consists of 4,705 species across 14 constituent timetrees, including a backbone timetree (Figure 5). This collection of timetrees was combined using the supertree construction method with parameters set as described above. In of Álvarez-Carretero et al. (2022), 13 constituent timetrees were attached to the corresponding node in the 72-species mammal timetree (backbone phylogeny) to assemble the 4,705 mammal species timetree. Therefore, we used the same set of trees they used to construct their 4,705 species timetree.

#### 2.3.3 Simiiformes dataset

**FIGURE 6:**
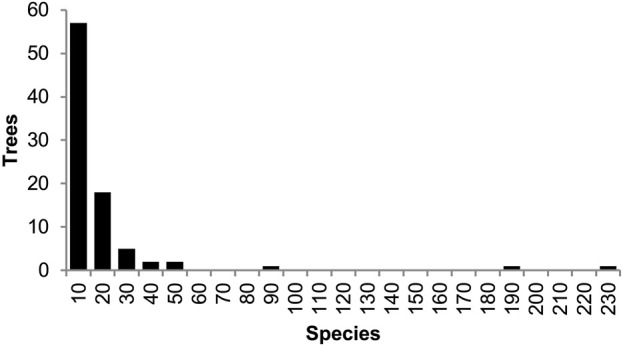
Distribution of the number of species present among 87 phylogenies of apes and monkeys (Simiiformes) from the TimeTree database. Across all trees, 270 total species were represented, with a median of seven species (2.6%) per timetree.

We further assessed the performance of Chrono-STA in an empirical context using data from TimeTree.org, which maintains a database of 4,185 timed molecular phylogenies (Figure 6). Given that the apes and monkeys (Simiiformes) are a well-studied group with numerous, occasionally conflicting, phylogenetic hypotheses, we selected this clade for our test (Craig et al., 2023). Starting from the full TimeTree database, we first selected only those trees containing at least two species-level OTUs representing the Simiiformes (enough for a single divergence time). This left us with 87 phylogenies of apes and monkeys ranging in size from three to 230 species (median seven, or 2.6% of the 270 total species included). Second, we pruned these phylogenies to include only those simiiform species with their names included in the NCBI taxonomy database in order to remove any non-simiiform taxa included by the original authors of these phylogenies. Third, to facilitate the Chrono-STA method, we took the optional step of adding two distant root species to each phylogeny: the chicken (*Gallus gallus*) with a divergence time from all Simiiformes of 319 Ma and the zebrafish (*Danio rerio*) at 429 Ma, using the inferred median time from the TimeTree database for both. By unambiguously rooting all trees, we avoid cases where Chrono-STA fails to find overlap without needing to assume any evolutionary relationships within the target clade. If the input phylogenies did lack sufficient overlap to construct a meaningful consensus in this case, Chrono-STA would be able to complete the run, but the resulting topology would be wildly inaccurate and differ substantially from other consensus trees like TimeTree. Thus, we are able to infer the accuracy of the Chrono-STA phylogeny by topological comparison to both the original input phylogenies and to the literature consensus phylogeny from TimeTree.

## 3 Results

### 3.1 Accuracy of constructed supertrees from simulated datasets

We first assessed the performance of Chrono-STA for the simulated data (Figure 3). Five of the six timetrees in each of the three collections had excellent taxonomic coverage within clades, but only a limited overlap with other timetrees. 73% species occur in just one of five trees, while only one species is common to all the timetrees. This design mimics empirical phylogenies which often specialize on given clades. Individual phylogenies in each collection differ in topology and times, because every timetree was inferred independently from a simulated multispecies alignment, as described in the Material and methods (Section 2.3.1).

**FIGURE 7:**
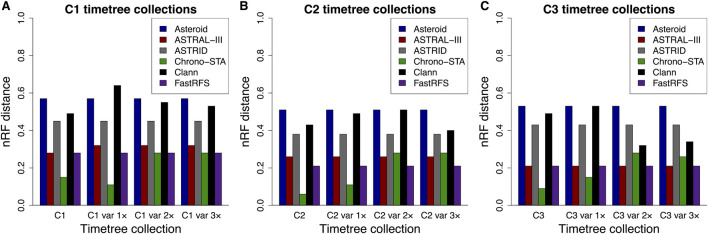
Normalized Robinson-Foulds (nRF) distances between the model timetree (reference tree) and the generated supertree are presented across timetree collections: **(A)** C1, **(B)** C2, and **(C)** C3. Results are shown for Asteroid (black), ASTRAL-III (red), ASTRID (grey), Chrono-STA (green), Clann (black), and FastRFS (purple). Var 1×, var 2×, and var 3× denote simulated node time variance increments of one, two, and three times the original variance, applied to the constituent timetree node times in C1, C2, and C3.

On average, Chrono-STA produced a supertree whose phylogeny agreed 90% with the reference tree, i.e., nRF = 0.1, from all three collections of timetrees (Figure 7). Therefore, Chrono-STA can work well for datasets with limited overlaps among major groups of taxa. FastRFS and ASTRAL-III achieved an average nRF of 0.23 and 0.25 respectively, which was twice as inaccurate as Chrono-STA. Other methods performed worse, with an average nRF of 0.42 for ASTRID, 0.47 for Clann, and 0.54 for Asteroid. Overall, these results suggest that the inclusion of chronology while combining phylogenies can produce higher accuracies when species overlaps are limited. The incorporation of the time dimension is a fundamental unifying factor, which other methods do not use as effectively as Chrono-STA.

Furthermore, we evaluated the impact of uncertainty in node times derived from constituent timetrees on the inferred supertree topology by using timetree collections C1–C3, with increasing node time variance of the constituent timetrees set to one (var 1×v), two (var 2×v), and three (var 3×v) times. We found that Asteroid, ASTRAL-III, ASTRID, and FastRFS are insensitive to node time uncertainty from constituent timetrees, with topological errors remaining the same under all conditions. However, Chrono-STA can be sensitive to extreme variance in the node times of the constituent timetrees. For C1–C3 var 1×, the average nRF increased slightly from 0.1 to 0.12, while for C1–C3 2× and C1–C3 var 3×, it rose to 0.28. For greater precision, we quantified the percentage time difference for each node in the corresponding timetrees between collections C1–C3 and their variants: C1–C3 var 1×, and C1–C3 var 3×fold variance increase, 21% with a twofold increase, and 28% with a threefold increase. The nRF also increases, from an average of 0.10 in C1–C3 to 0.12 in C1–C3 var 1× and 0.28 in C1–C3 var 2× but remains unchanged in C1–C3 var 3×.

**FIGURE 8:**
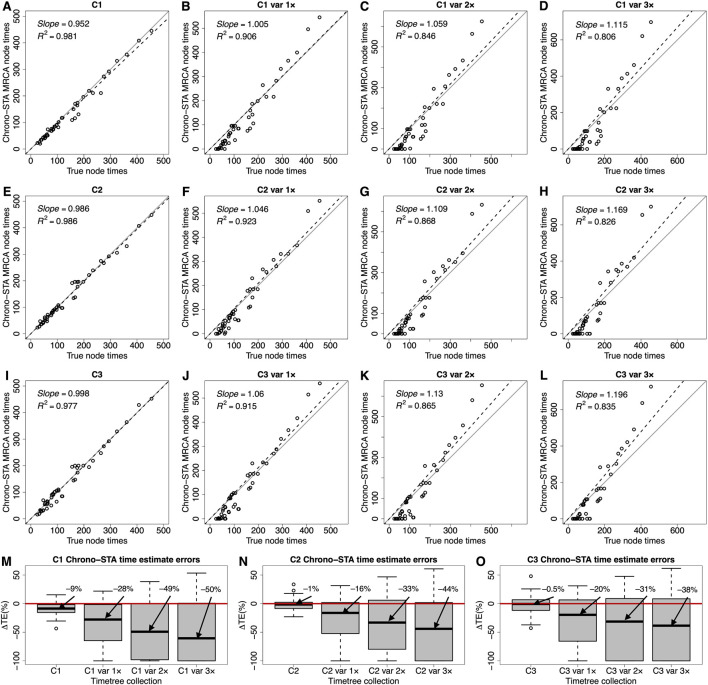
**(A–L)** Comparison of time estimates obtained by using Chrono-STA with the true node times for collections C1-C3. The slope and coefficient of determination (*R* ^2^) for the linear regression through the origin are shown. The black dashed lines represent the best-fit linear regression through the origin. The solid gray line represents equality between estimates. **(M–O)** Distributions of the differences between Chrono-STA node times and true node times (ΔTEs). The black horizontal line represents the median value. Var 1×, var 2×, and var 3× denote simulated node time variance increments of one, two, and three times the original variance, applied to the constituent timetree node times in C1, C2, and C3. For Chrono-STA, we used the estimated node times for the MRCA of all the sets of taxa in the model timetree (reference tree).

Chrono-STA produces divergence times along with the phylogeny. So, we compared the time estimates from the inferred Chrono-STA supertree with those in the reference tree. We used the Chrono-STA node times for the MRCA of all the sets of taxa in the reference tree. Chrono-STA generated node times highly consistent with those of the reference tree (Figure 8), with slope and *R* ^2^ values approaching 1.0 across timetree collections C1-C3. We also quantified the accuracy of Chrono-STA by computing the difference between the estimated MRCA node times and the true node times. The median ΔTE was low for the three datasets (Figure 8D), at −9% for C1, –1% for C2, and –0.5% for C3.

We evaluated the impact of increasing levels of node time variance in the constituent timetrees on Chrono-STA time estimates and found a considerable effect. The slope and *R* ^2^ values consistently deteriorated as the variance increased by one (var 1×), two (var 2×), and three (var 3×) times (Figures 8A–L). The distribution of ΔTEs were considerably wider, and the median was significantly higher for these timetree collections (Figures 8M–O). The notable impact of node time variance on Chrono-STA time estimates implies that the simulated variance was substantial. Nonetheless, Chrono-STA demonstrates the ability to generate reliable supertree topologies under such challenging conditions.

### 3.2 Accuracy of constructed supertrees from empirical datasets

#### 3.2.1 Mammal dataset

**FIGURE 9:**
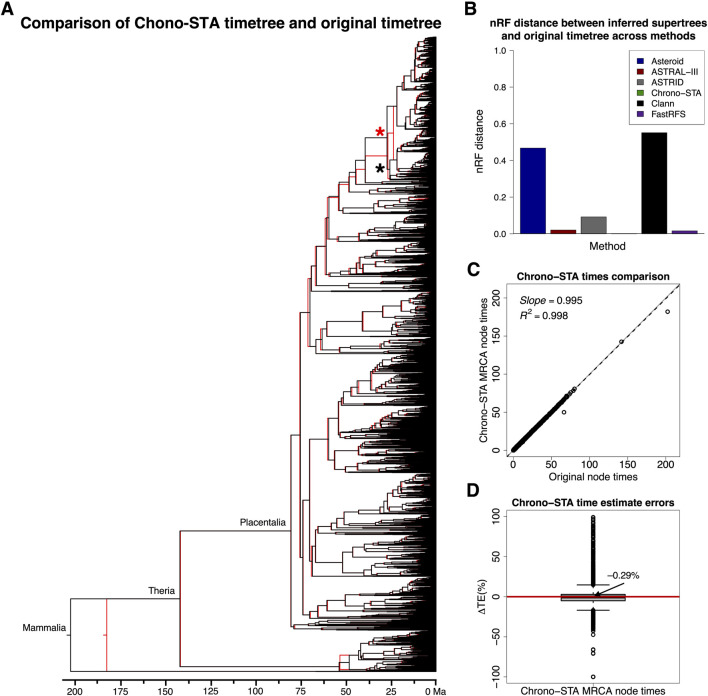
**(A)** Comparison of the 4,705 mammal species timetree (black) from Álvarez-Carretero et al. (2022) and Chrono-STA supertree (red). The supertree was constructed by combining 14 constituent timetrees including a backbone timetree. **(B)** nRF distances between the original timetree and the generated supertree for the 4,705 mammal species dataset (Álvarez-Carretero et al., 2022) for Asteroid (black), ASTRAL-III (red), ASTRID (grey), Chrono-STA (green), Clann (black), and FastRFS (purple). The supertree was constructed by combining 14 constituent timetrees including a backbone timetree. The topological differences are marked with red and black asterisks. **(C)** Comparison of original and Chrono-STA time estimates. The slope and coefficient of determination (*R* ^2^) for the linear regression through the origin are shown. The black dashed lines represent the best-fit linear regression through the origin. The solid gray line represents equality between estimates. **(D)** Distribution of ΔTEs between the estimated and original node times. For Chrono-STA, we used the estimated node times for the MRCA of all the sets of taxa in the original timetree.

We validate these trends observed in simulated data by analyzing the large empirical dataset of Álvarez-Carretero et al. (2022), containing 4,705 mammal species across 14 taxonomically restricted timetrees (Figure 5). Chrono-STA assembled these timetrees into a supertree that was identical in topology to that published by Álvarez-Carretero et al. (2022) (Supplementary Figure S1), except for a single internal branch which shifted to its sister clade, indicated by a red and a black asterisk in Figure 9A. The nRF for Chrono-STA was 0.0002 (Figure 9B). FastRFS performed the second best generating a supertree with 75 topological differences (nRF = 0.016) from the reference tree (Supplementary Figure S2), ASTRAL-III performed the third best generating a supertree with 96 topological differences from the reference tree (Supplementary Figure S3), which is almost 200-times worse than Chrono-STA (nRF = 0.02). No other tested method performed well (Figure 9B). The ASTRID supertree had 430 differences from the reference tree (Supplementary Figure S4; nRF = 0.09), while Asteroid had 2,198 differences (Supplementary Figure S5) and achieved an nRF of 0.47. Clann was outperformed by all the other methods, generating a supertree with 2,592 topological differences from the reference tree, achieving an nRF distance of 0.55 (Supplementary Figure S6). Therefore, as in simulation, Chrono-STA produced reliable supertrees from empirical datasets comprised of highly taxonomically restricted timetrees.

Chrono-STA recovered the correct node times as well, as they closely aligned with those of the original timetree, except for the Mammalia Chrono-STA node age (Figure 9A). This discrepancy arose because, in Chrono-STA, the calculation for that node time involved averaging across the 14 constituent timetrees, whereas in the original timetree, it represented the mammalian node time estimated independently for the backbone timetree. The slope and *R* ^2^ values were nearly 1.0 (Figure 9C), and the median ΔTE was −0.29% (Figure 9D).

#### 3.2.2 Simiiformes dataset

We further used Chrono-STA to infer a phylogenetic consensus of 270 ape and monkey (simiiformes) species from a sample of 87 published molecular trees in the TimeTree database. These trees ranged from three to 230 species, with a median size of just seven simiiform species (2.6% of the total), meaning that taxonomic overlap would be sparse in many places, and variances in estimated divergence times for shared nodes would be unavoidable.

To quantify the performance of Chrono-STA, we compared the nRF distance between the published input tree and the matching subset of our inferred Chrono-STA tree, under the assumption that if Chrono-STA is accurately inferring a topology reflective of the input trees, then there should be minimal difference between these two. We further compared the performance of Chrono-STA to that of the hierarchical average linking (HAL) algorithm employed by TimeTree. HAL differs from Chrono-STA in that it uses a hierarchical clustering algorithm to resolve polytomies on a topological backbone, in this case provided by the NCBI taxonomy database. For this comparison, we again took nRF distances between the input trees and the subset of the published fifth edition of the TimeTree of Life consensus phylogeny. We propose that if the Chrono-STA tree, which was inferred without the use of a phylogenetic backbone, does not significantly differ from the HAL tree, which used the NCBI backbone, then we can conclude that Chrono-STA does indeed release the constraint of assuming a backbone topology when analyzing sparse empirical data.

When comparing the study trees to corresponding subsets of both the Chrono-STA and HAL trees, the Chrono-STA tree had a mean nRF distance of 0.17 across all comparisons between a study tree and the corresponding subset of the supertree, compared to 0.17 for the HAL tree (paired t-test P > 0.10). Among the study trees, three contained a single polytomy each, while the final Chrono-STA tree was fully resolved, with no polytomies at all. By contrast, 24 subsets of the HAL tree included polytomies, for a total of 174 total species descending from polytomies across all subsets. Thus, the HAL tree was not significantly more accurate than the Chrono-STA tree with respect to the input study trees, but it was substantially less powerful in its ability to resolve polytomies. This means that the phylogenetic backbone required by HAL does not improve the accuracy of the inferred topology compared to Chrono-STA, but in fact limits its ability to resolve divergences, likely due to taxonomic disagreements with the input tree set.

## 4 Discussion

We found that the new Chrono-STA approach can excel in building timetrees in cases where missing data are not randomly distributed among trees but instead concentrated in certain clades (phylogenetically restricted). This better reflects the current state of the corpus of published literature, as researchers tend to specialize in certain families and genera and assemble detailed phylogenies of phylogenetically restricted groups. For such data with sparse species overlaps, the use of chronological information in times can help build better supertrees. While input timetrees can be generated using Bayesian or maximum likelihood methods, in Chrono-STA timetrees are combined using our pairwise average linkage approach where the act of clustering and subsequent backpropagation in each iteration inherently increases the completeness of the distance matrices at each step. In addition to this fundamental conceptual reason for a distance-based phylogenetic method, average linkage has practical benefits in that it is computationally efficient, enabling faster iterative analyses, especially with large datasets. Such a method would not be possible in a reasonable timeframe for most datasets using Bayesian or ML methods due to their high computational demands and long processing times.

ASTRAL, Asteroid, and Astrid were developed for gene tree species tree reconciliation, based on the implicit assumption that individual gene trees typically represent a subset of the species under study rather than predominantly disjoint subsets of distinct species. Consequently, these methods are not designed to function as classic supertree approaches. The performance improvement we observe from Chrono-STA as compared to the gene tree reconciliation approaches in building a supertree from phylogeny collections with phylogenetically restricted sparsity is likely due to the incorporation of time information. However, there was a large difference between the performance of ASTRAL-III and other methods (Asteroid and ASTRID). This difference likely arises from fundamental conceptual differences between them. ASTRAL-III (Zhang et al.,2018) combines phylogenies using a quartet-puzzling approach in which each constituent phylogeny is represented in batches of four taxa, and then the relative frequencies with which each of these quartets occur across all phylogenies are used to build the consensus supertree. In contrast, other Asteroid and ASTRID use distance between taxa in constituent phylogenies in the units of the number of intervening nodes or edges between taxa. When taxa are missing in some phylogeny, they impute missing distances statistically and then build a global matrix of pairwise distances to apply distance-based approaches, such as the Neighbor-Joining (Saitou and Nei, 1987), to construct a supertree.

Relative performance of many different versions of the imputation and quartet puzzling approaches have been examined for gene tree reconciliation with and without missing data (Cao and Nakhleh, 2019; Rabiee et al., 2019; Zhang et al., 2020; Zhang and Mirarab, 2022; Liu and Warnow, 2023). The general conclusion seems to be that they perform well and similarly. This is supported by our results, where ASTRAL-III consistently performed second-best after Chrono-STA, followed by a considerable gap in performance between ASTRAL-III and methods that used imputation to overcome missing data like Asteroid and ASTRID. This is not surprising because the reliability of any imputation is expected to be proportional to the amount of data available, resulting in more error when data are sparser. Furthermore, in cases of phylogenetically restricted sparsity, which, again, reflects the literature, this imputation is likely especially unreliable on a clade-by-clade basis, where there may be significantly less data than the matrix-wide average for some poorly studied clades. This would explain why ASTRAL-III, a quartet-puzzling approach that does not rely on imputation, achieves better accuracy than other methods except for Chrono-STA.

The FastRFS analysis implemented in this study used ASTRAL machinery and outperformed the ASTRAL-III method implemented here. This is likely because FastRFS addresses missing data by focusing on shared taxa between input trees, using these taxa to calculate RF distances and guide supertree construction, while excluding missing taxa to avoid artificial conflicts. However, the method relies on sufficient taxonomic overlap, making it less effective for sparse overlaps. In such cases, it may provide reduced resolution for limited overlapping taxa, face challenges handling conflicts in overlapping regions, and potentially introduce bias toward well-sampled areas.

Clann, a supertree method, has difficulties with limited taxonomic overlap, as the four-point condition tends to produce less accurate estimates when there is insufficient shared information across constituent trees. Sparse overlap increases the likelihood of errors in inferred distances, which can prevent the heuristic search from converging on an accurate supertree. Furthermore, the search space becomes harder to navigate effectively without sufficient taxonomic overlap to guide optimization.

We have demonstrated that Chrono-STA is capable of integrating timetrees despite limited taxonomic overlap by incorporating phylogenetic temporal information. Furthermore, Chrono-STA is highly robust to variance in node times, as demonstrated in our tests of induced variance, where node times were modified by 1×, 2×, and 3×. In this test, we found that our inferred divergence times were largely robust to variance in the input timetrees, so while summarizing this variance in the final timetree will be an aim of future work, we are confident that the method is robust to such error. However, Chrono-STA may exhibit some sensitivity to discrepancies in time estimates across constituent timetrees and currently does not directly propagate uncertainty at each node from constituent timetrees into the final supertree. Incorporating uncertainty estimation into the framework could be a valuable direction for future work.

Therefore, Chrono-STA occupies a unique niche as a supertree method tuned for the high degree of taxonomic incompleteness we observe in real empirical datasets. While any approach is likely to perform well in cases with many constituent trees and a high degree of overlap, Chrono-STA is ideally suited to building a phylogenetic consensus across higher taxonomic levels, while biodiversity experts often prioritize depth within their chosen clade of interest rather than breadth across the tree of life. This makes Chrono-STA an attractive approach for reconstructing the history of life on Earth.

## Data Availability

The datasets presented in this study can be found in online repositories. The names of the repository/repositories and accession number(s) can be found below: https://github.com/josebarbamontoya/chrono-sta.
