# Supplementary material for: Integrating phylogenies with chronology to assemble the tree of life
Source: Front Bioinform. 2025 Apr 30;5:1571568. doi: 10.3389/fbinf.2025.1571568 (PMC12075222; doi:10.3389/fbinf.2025.1571568)
Supplement: Supplementary file 1 [file DataSheet1.pdf]

Integrating phylogenies with chronology to assemble the Tree of Life

Supplementary Material

Jose Barba-Montoya[1,2], Jack M Craig[2,3], Sudhir Kumar[2,3*]

[1]Richard Gilder Graduate School, American Museum of Natural History, New York, NY.

[2]Institute for Genomics and Evolutionary Medicine, Temple University, Philadelphia, PA. USA.

[3]Department of Biology, Temple University, Philadelphia, PA. USA.

*** Correspondence:**
Sudhir Kumar
s.kumar@temple.edu

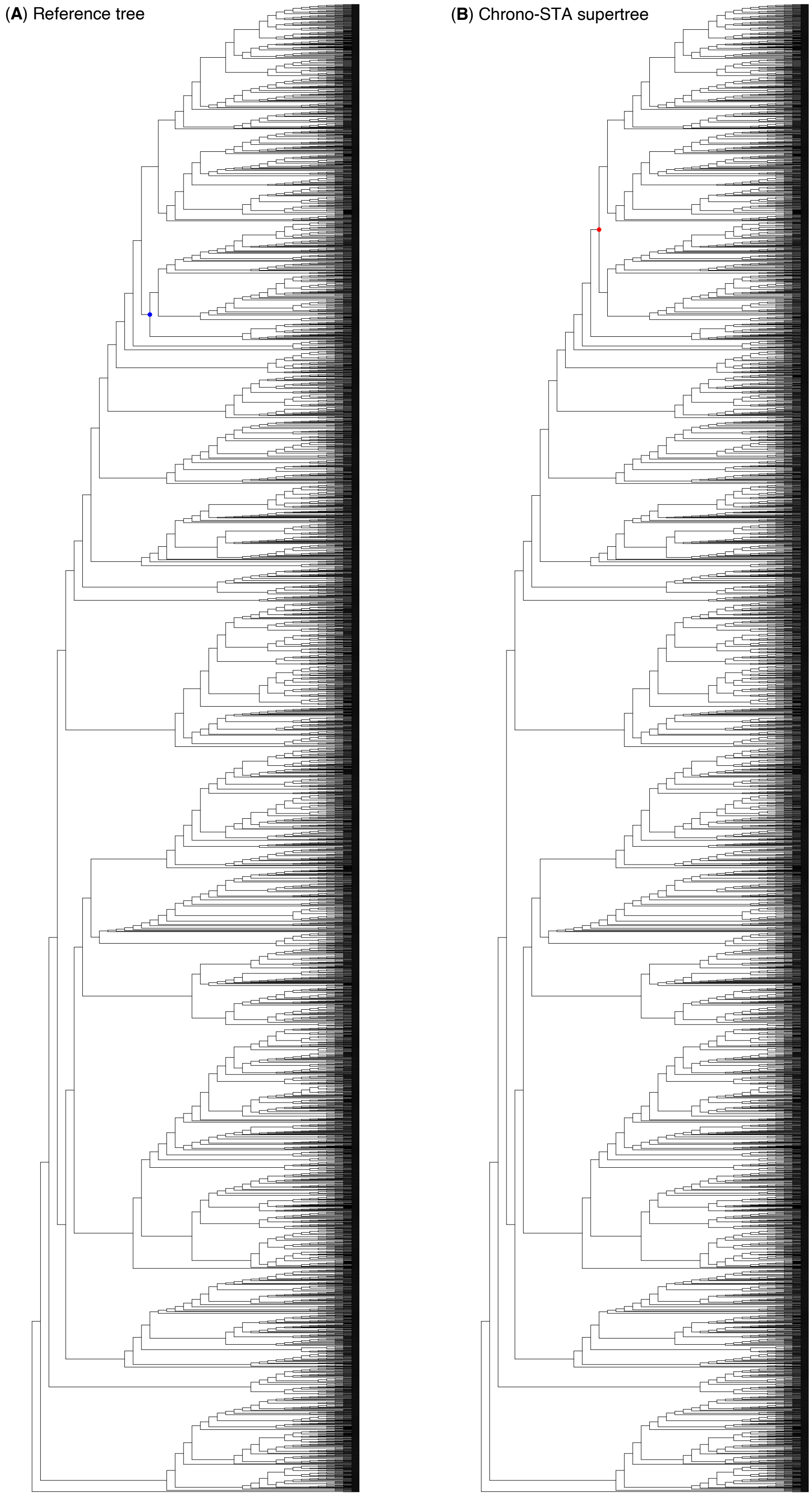

Supplementary Figure 1. Comparison of (A) the mammal timetree (Álvarez-Carretero *et al.*, 2022). consisting of 4,705 species (reference tree) and (B) the Chrono-STA supertree. Red dots represent clades absent in (A), and blue dots represent clades absent in (B).

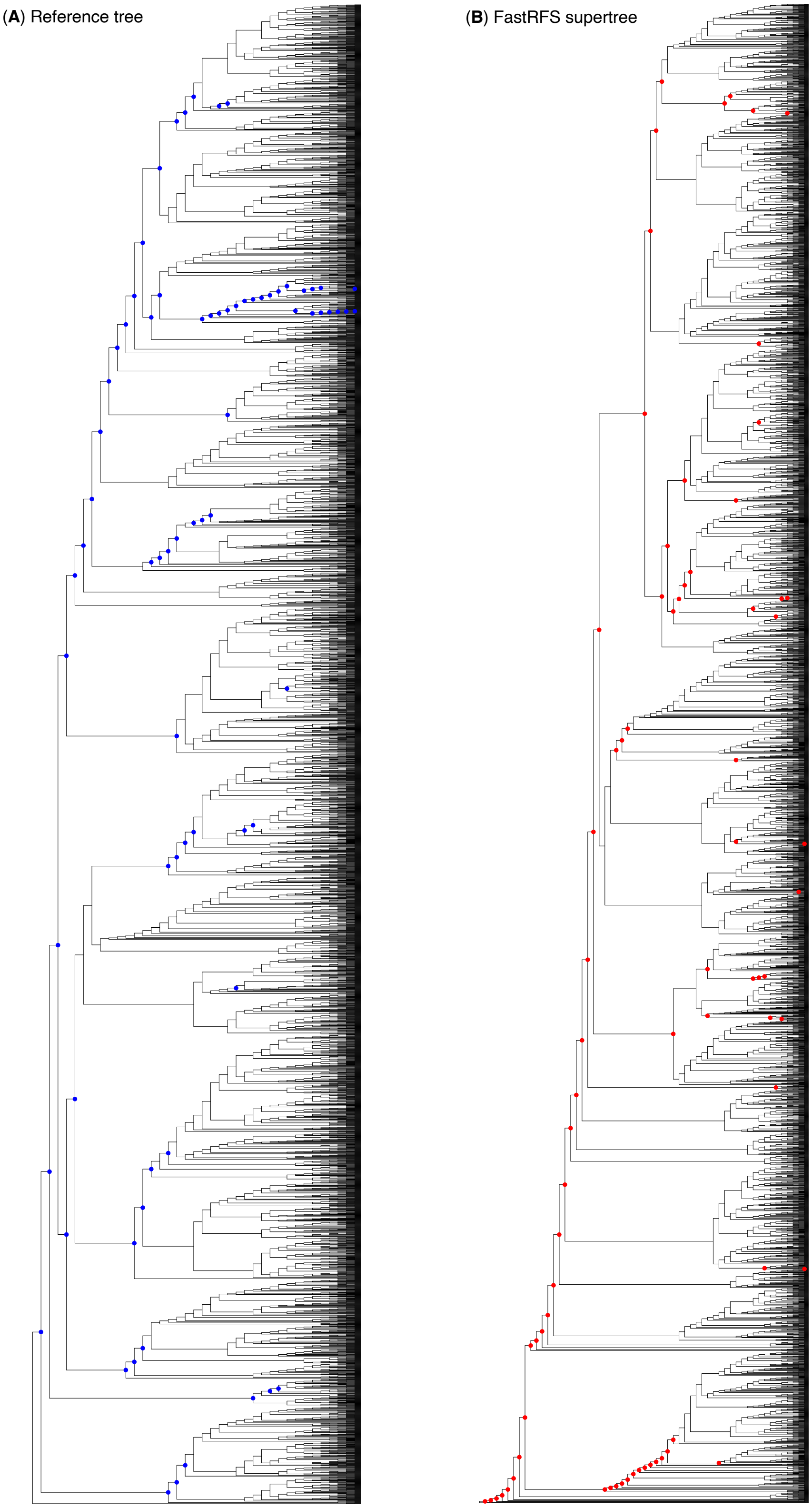

Supplementary Figure 2. Comparison of (A) the mammal timetree (Álvarez-Carretero *et al.*, 2022). consisting of 4,705 species (reference tree) and (B) the FastRFS supertree. Red dots represent clades absent in (A), and blue dots represent clades absent in (B).

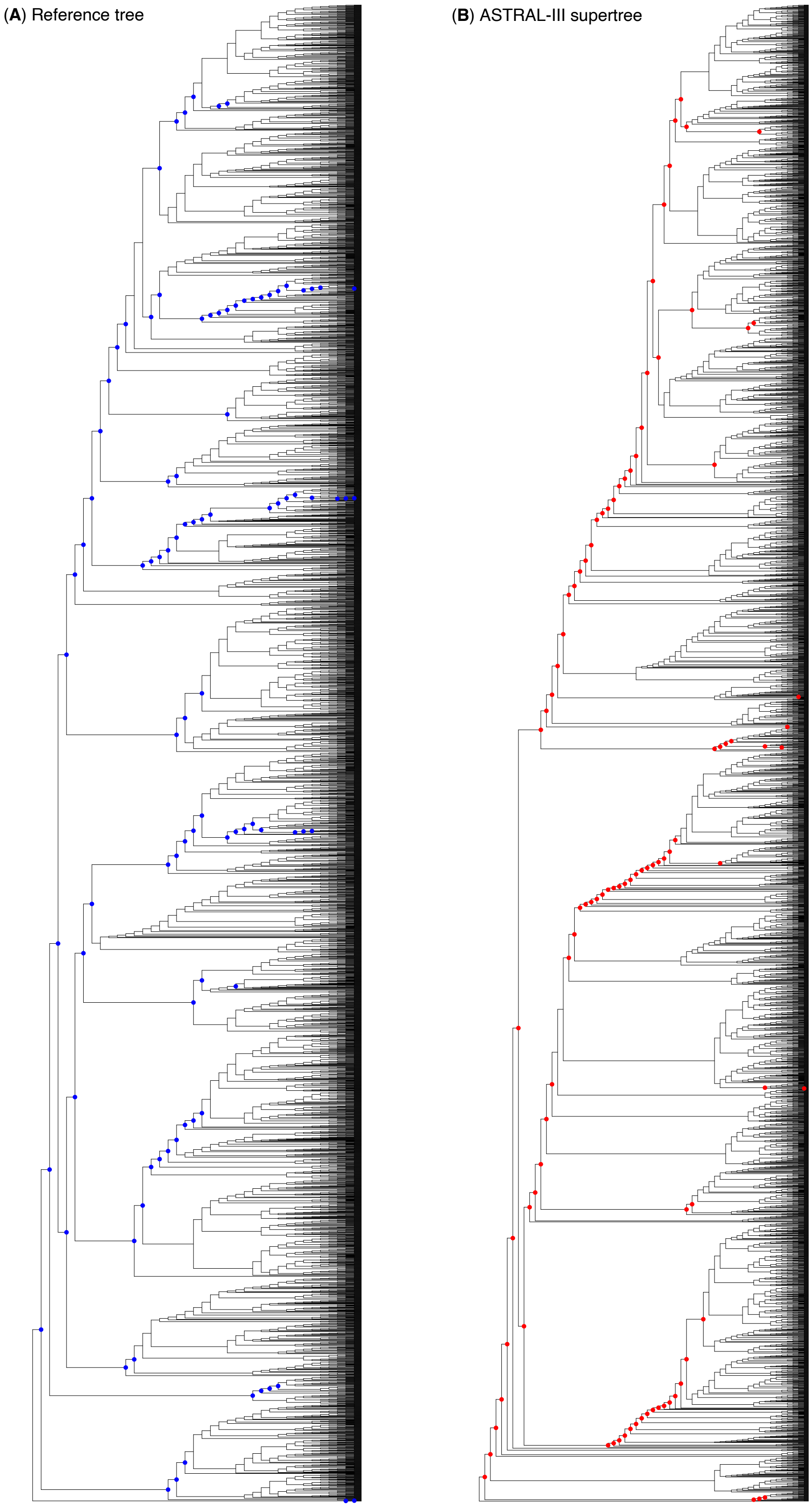

Supplementary Figure 3. Comparison of (A) the mammal timetree (Álvarez-Carretero *et al.*, 2022). consisting of 4,705 species (reference tree) and (B) the ASTRAL-III supertree. Red dots represent clades absent in (A), and blue dots represent clades absent in (B).

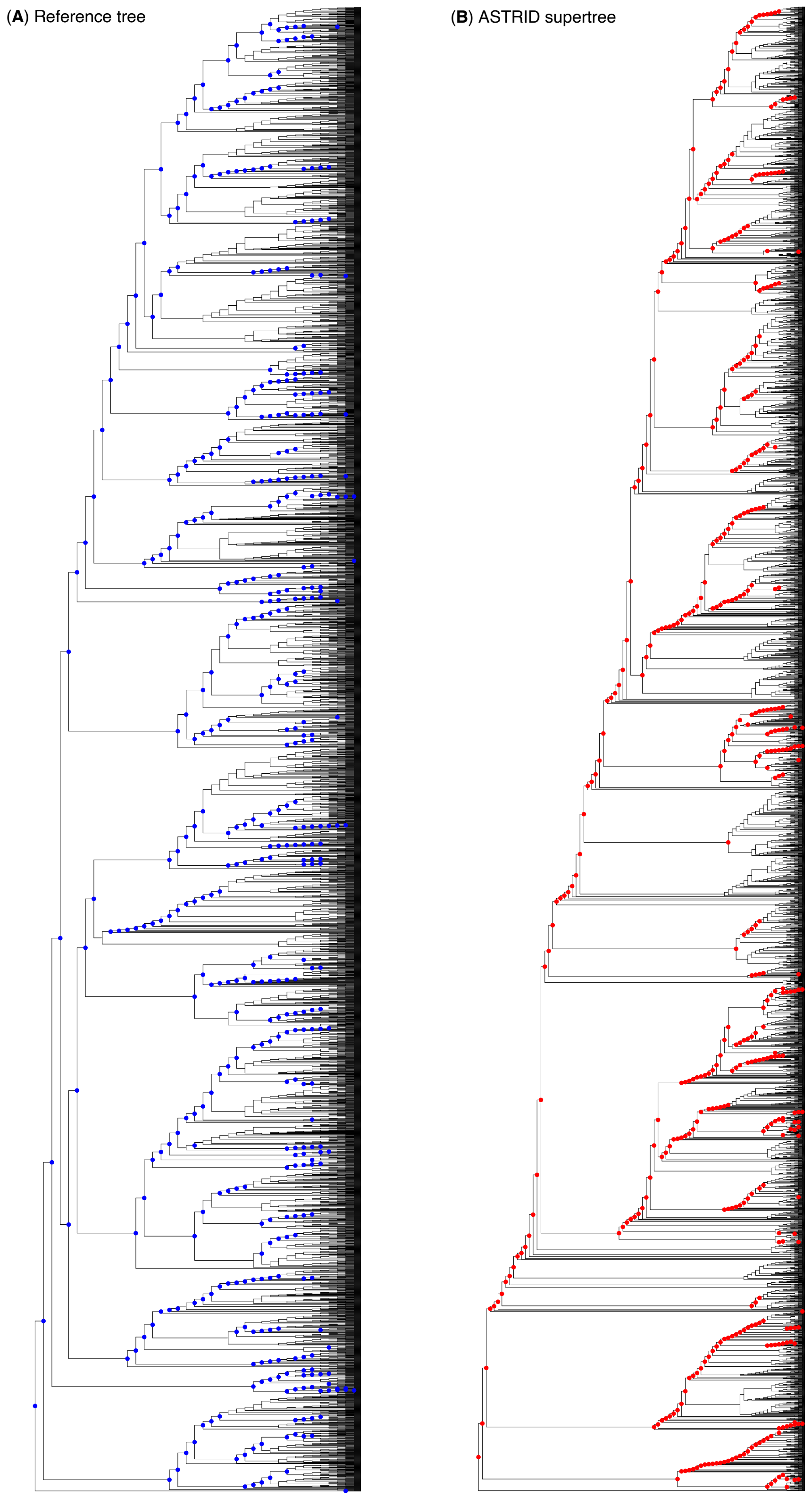

Supplementary Figure 4. Comparison of (A) the mammal timetree (Álvarez-Carretero *et al.*, 2022). consisting of 4,705 species (reference tree) and (B) the ASTRID supertree. Red dots represent clades absent in (A), and blue dots represent clades absent in (B).

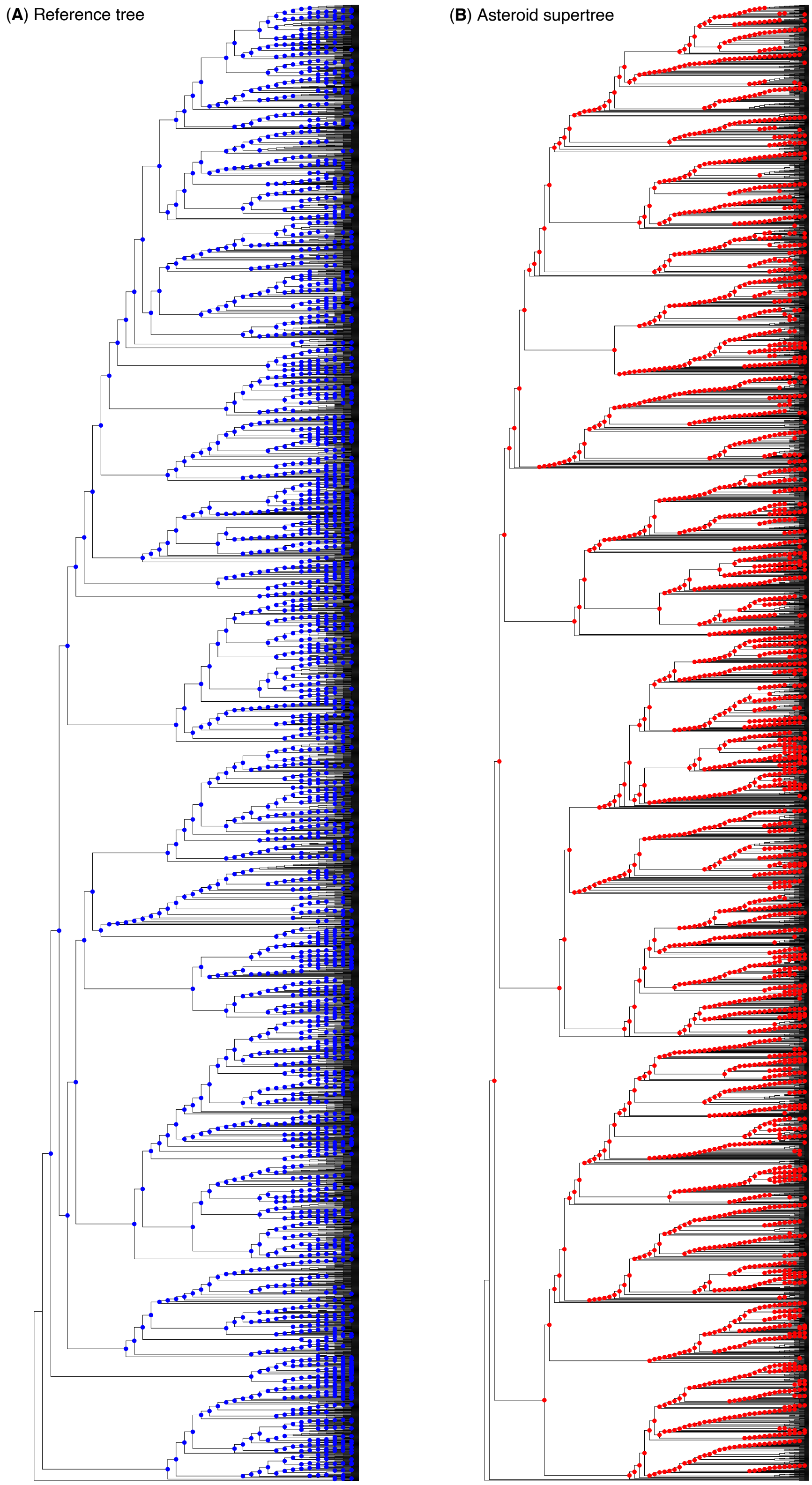

Supplementary Figure 5. Comparison of (A) the mammal timetree (Álvarez-Carretero *et al.*, 2022). consisting of 4,705 species (reference tree) and (B) the Asteroid supertree. Red dots represent clades absent in (A), and blue dots represent clades absent in (B).

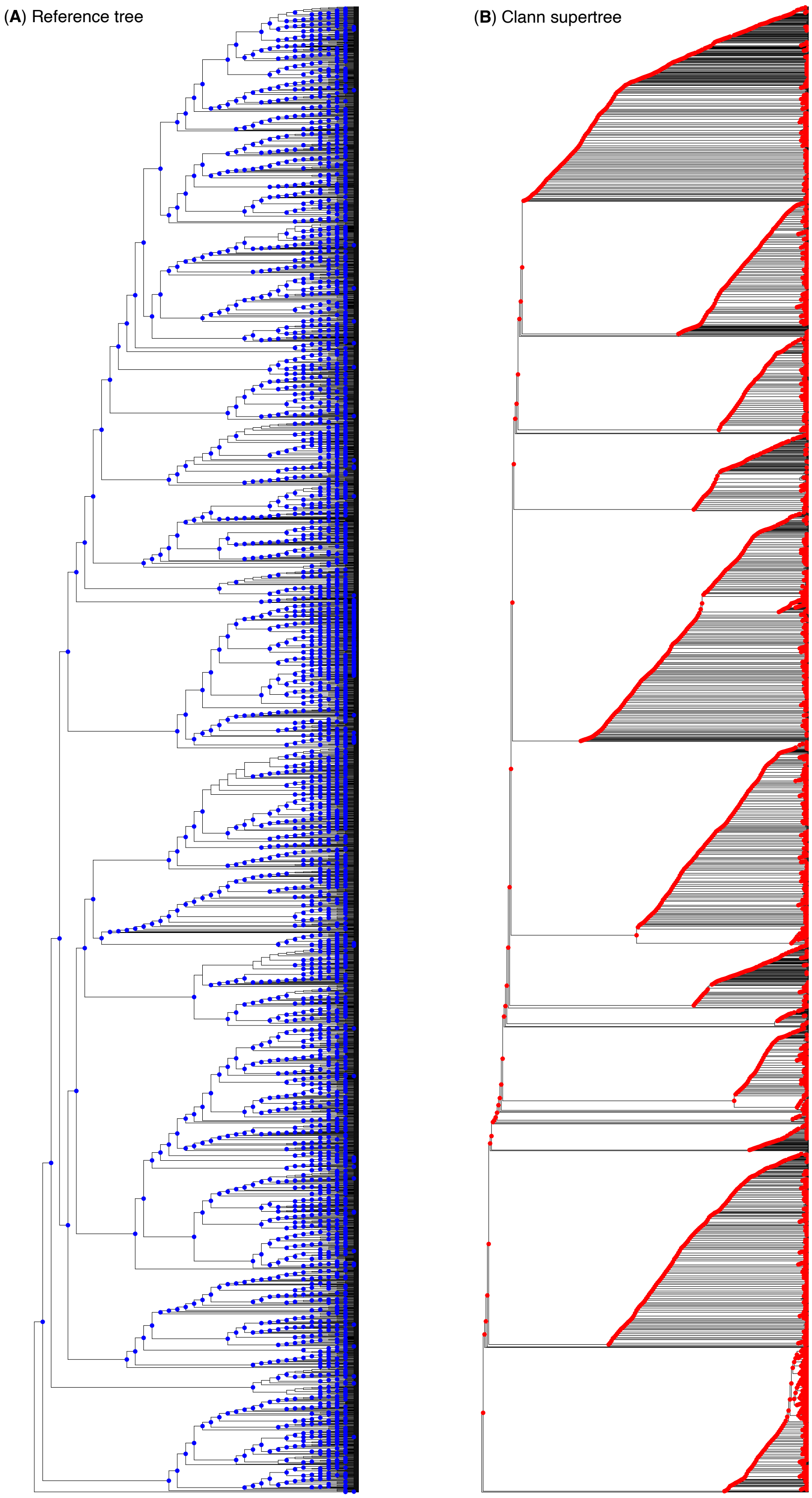

Supplementary Figure 6. Comparison of (A) the mammal timetree (Álvarez-Carretero *et al.*, 2022). consisting of 4,705 species (reference tree) and (B) the Clann supertree. Red dots represent clades absent in (A), and blue dots represent clades absent in (B).
